# Changes in Prefrontal Cortical Activity During Walking and Cognitive Functions Among Patients With Parkinson's Disease

**DOI:** 10.3389/fneur.2020.601686

**Published:** 2020-12-10

**Authors:** Maud Ranchet, Isabelle Hoang, Maxime Cheminon, Romain Derollepot, Hannes Devos, Stephane Perrey, Jacques Luauté, Teodor Danaila, Laurence Paire-Ficout

**Affiliations:** ^1^TS2-LESCOT, Univ Gustave Eiffel, IFSTTAR, Univ Lyon, Lyon, France; ^2^Service de Médecine Physique et de Réadaptation Neurologique, Hôpital Henry-Gabrielle, Hospices Civils de Lyon, Lyon, France; ^3^Department of Physical Therapy and Rehabilitation Science, School of Health Professions, The University of Kansas Medical Center, Kansas City, KS, United States; ^4^EuroMov Digital Health in Motion, Univ Montpellier, IMT Mînes Ales, Montpellier, France; ^5^Inserm UMR-S 1028, CNRS UMR 529, ImpAct, Center de Recherche en Neurosciences de Lyon, Université Lyon-1, Bron, France; ^6^Université de Lyon, Université Claude Bernard Lyon 1, Lyon, France; ^7^Center de Neurosciences Cognitives, Service de Neurologie C, Hôpital Neurologique Pierre Wertheimer, Hospices Civils de Lyon, Université Claude Bernard Lyon I, Lyon, France

**Keywords:** Parkinson's disease, fNIRS, walking, cognition, dual-task walking, gait

## Abstract

**Background:** Walking becomes more and more degraded as Parkinson's Disease (PD) progresses. Previous research examined factors contributing to this deterioration. Among them, changes in brain cortical activity during walking have been less studied in this clinical population.

**Objectives:** This study aimed to: (1) investigate changes in dorsolateral prefrontal cortex (DLPFC) activation during usual walking and dual-task walking conditions in patients with PD; (2) examine the association between cortical activity and behavioral/cognitive outcomes; and (3) explore which factors best predict increased activation of the DLPFC during usual walking.

**Methods:** Eighteen patients with early stage PD and 18 controls performed 4 conditions: (1) standing while subtracting, (2) usual walking, (3) walking while counting forward, and (4) walking while subtracting. Cortical activity in DLPFC, assessed by changes in oxy-hemoglobin (ΔHbO_2_) and deoxy-hemoglobin (ΔHbR), was measured using functional near infrared spectroscopy (fNIRS). Gait performance was recorded using wearables sensors. Cognition was also assessed using neuropsychological tests, including the Trail Making Test (TMT).

**Results:** DLPFC activity was higher in patients compared to controls during both usual walking and walking while subtracting conditions. Patients had impaired walking performance compared to controls only during walking while subtracting task. Moderate-to-strong correlations between ΔHbO_2_ and coefficients of variation of all gait parameters were found for usual walking and during walking while counting forward conditions. Part-B of TMT predicted 21% of the variance of ΔHbO_2_ during usual walking after adjustment for group status.

**Conclusions:** The increased DLPFC activity in patients during usual walking suggests a potential compensation for executive deficits. Understanding changes in DLPFC activity during walking may have implications for rehabilitation of gait in patients with PD.

## Introduction

Walking in everyday life may be a complex task for individuals with Parkinson's Disease (PD). Patients with PD have gait problems, such as reduced gait speed and step length (1), that worsen as the disease progresses. These gait problems may be exacerbated during dual-task (DT) walking combining both motor and cognitive activities. This may lead to a higher risk of falls (2), reduced mobility and quality of life (3).

Previous studies have investigated behavioral (e.g., cognitive, motor) and neurophysiological measures [e.g., functional magnetic resonance imagery (fMRI), electroencephalography (EEG)] that could predict impaired walking or falls risk in patients with PD (2, 4–6). Among neurophysiological measures, changes in brain cortical activity during walking have been less studied in patients with PD (7). Different neurophysiological techniques have been proposed to better examine changes in brain activation during walking in PD.

Evidence from functional magnetic resonance imagery (fMRI) suggest that patients with PD had higher activation in frontal, parietal, temporal, and occipital lobes than healthy older adults during imagined usual walking (6). The increased cortical activation in patients with PD may reflect a compensatory mechanism to overcome inefficient neural activation. Although fMRI has adequate spatial resolution, changes in brain activation during real walking conditions in patients with PD are unfeasible to capture in such neuroimaging studies. Portable electroencephalography (EEG) or functional near-infrared spectroscopy (fNIRS) techniques have allowed investigation of cortical activity during real-time walking (8). Using EEG, specific changes in electrical brain activity during DT walking were found in patients with PD (9). Patients with PD showed reduced P300 amplitude during DT walking compared to standing. This reduced P300 amplitude reflects unsynchronized activation of various resources (lower timing and coordination between different brain regions), suggesting less neural recruitment during DT walking. EEG has a good temporal resolution compared to other neuroimaging techniques. However, the spatial resolution of EEG is relatively low compared to fNIRS and neck muscles as well as artifacts such as eye movements can affect the quality of the recordings. With recent advances in technology, fNIRS allows an indirect evaluation of brain activation by measuring changes in brain blood oxygen levels. The fNIRS technique emits light with different wavelengths that is partly absorbed by the chromophores such as oxygenated and deoxygenated hemoglobin (HbO_2_ and HbR, respectively). Increased brain activation induces an intensified blood flow in the active brain regions leading to an increase in HbO_2_ and decrease of HbR (10).

To date, few studies have explored cortical activity during walking in patients with PD, using fNIRS (6, 11–18). Some studies reported both HbO_2_ and HbR (12) and only one study used reference channels to correct for superficial hemodynamic interferences (15). Furthermore, the associations between neurophysiological and behavioral outcomes during usual and DT walking tasks were poorly understood (19) in patients with PD (20, 21). Under challenging tasks [e.g., obstacle negotiation (20) or DT walking while subtracting (12, 14)], patients showed an increased activation of prefrontal cortex (PFC) that may affect walking performance. An explanation to this increased activation in the PFC could be that basal ganglia dysfunction leads to reduced movement automaticity, which increases reliance on executive resources to control movements. The PFC, specifically the DLPFC plays a major role in executive functions, essential for the management of cognitive functions including planning, working memory and cognitive flexibility (22). Compared to healthy older adults, patients with PD showed a higher activation in the PFC compared to controls, even during usual walking (12, 20). This may reduce the functional reserve needed during more demanding tasks which may contribute to high prevalence of falls and DT difficulties among patients with PD (6). The role of DLPFC activity during usual walking should therefore better be explored in individuals with PD.

The objectives of this study were (1) to investigate changes in DLPFC activation during usual walking and DT walking conditions in patients with PD compared to controls, (2) to examine the association between cortical activity and behavioral/cognitive outcomes, and (3) to explore which factors best predict increased activation of the DLPFC during usual walking.

## Materials and Methods

### Participants

Patients were recruited from the Henry-Gabrielle Hospital, Lyon and healthy older adults (controls) were recruited through advertisements between September 1, 2018 to December 17, 2019.

General inclusion criteria were: 50 years old and above and able to walk unassisted for at least 20 mins. All participants had normal-to-corrected vision. All participants had no other visual, neurological (other than PD for the PD group), internal or psychiatric conditions that may interfere with walking. Patients with PD were excluded if they had Montreal Cognitive Assessment (MOCA) scores ≤ 15, other parkinsonian syndromes, severe dyskinesia, deep brain stimulation, unpredictable motor fluctuations, or ocular diseases causing significant visual impairment.

Fear of falling was assessed by the Falls Efficacy Scale International (FES-I) and symptoms of depression were measured by the Beck Depression Inventory scale. Total daily levodopa-equivalent dose was calculated for each PD patient (23). All patients were medicated and tested in the “on” medication state. The “on” state was defined by a doctor (MC or TD) during the medical examination that preceded the experiment. Patients were tested between 1 and 3 h after taking their medication.

The study was approved by the French biomedical ethics committee on March, 9 2018 (Comité de Protection des Personnes Nord Ouest III Réf. CPP: 2018-01 N° ID RCB: 2017-A03187-46). Informed written consent was obtained from all participants.

### Protocol

**Figure 1:**
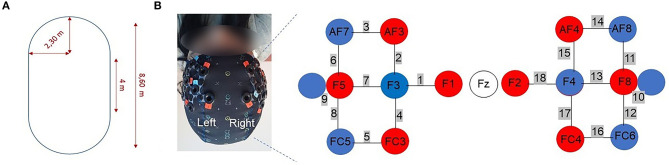
**(A)** Walking path configuration. **(B)** Optodes configuration. The right panels show the optodes with respect to the Fz locations of the international 10-10 system. Red circles represent the sources and blue circles represent the detectors. The numbers in gray represent the channels. Channels 9 and 10 represent the short separation channels.

Participants performed in a single session 4 conditions: (1) standing while subtracting, (2) usual walking, (3) walking while counting forward, and (4) walking while subtracting. In the standing while subtracting condition, participants were required to stand still while subtracting 7 to a 3-digit number out loud. In the usual walking condition, participants were asked to walk at their “normal pace.” In the walking condition while counting forward, they were instructed to walk while counting forward out loud from a random 3-digit number. In the walking condition while subtracting, they were asked to subtract 7 from a random 3-digit number out loud. No instructions about priorization were given for the DT walking conditions. Each condition included 5 trials of 30 s. The duration of rest periods between trials ranged from 25 to 35 s in order to avoid anticipation of block onset. During rest periods, participants were instructed to stand still quietly. During the standing while subtracting and the two DT walking conditions, cognitive performance was measured and defined as the number of correct operations. The order of each condition was randomized between participants. Each condition started with 45 s of standing quietly (resting state period), with the instruction to refrain from talking and moving the head. The walking path configuration was an oval shape whose dimensions are displayed in Figure 1A. In order to assist participants to walk on the path, there were markings on the floor. Participants were allowed to take breaks between conditions. Furthermore, all experiments took place in the morning, the fatigue being less pronounced than in the afternoon.

Gait was assessed using two wearable sensors placed on the shoes (Physiolog®5, Gait Up, Switzerland). Walking performance parameters included mean gait speed (m/s), cadence (step/min), stride length (m), gait cycle time (s), and their coefficient of variation (CV) calculated as (standard deviation/mean)^*^100. For each condition, gait outcome measures were averaged over the 5 trials.

Relative concentration changes of HbO_2_ and HbR (ΔHbO_2_ and ΔHbR in μmol/L, respectively) in the DLPFC were measured using a wireless continuous waves fNIRS device (NIRSport, NIRx Medical Technologies) with 16 optodes (8 sources and 8 detectors).

### Functional Near Infrared Spectroscopy

An increase of ΔHbO_2_ associated with a slight decrease of ΔHbR reflects a functional activation for the task (24). Fourteen optodes, corresponding to the 8 sources and 6 detectors separated by ~30 mm, were placed on the DLPFC according to the modified international EEG 10-10 system. Two short separation channels (channels 9 and 10, see Figure 1B) with an interoptode distance of 15 mm were used in order to account for peripheral tissue signals. The near infrared light was emitted by sources with wavelengths of 760 and 850 nm at a sampling rate of 7.81 Hz. Textile EEG caps in 3 different sizes (i.e., circumference of 54, 56, 58 cm) were used in order to fix the sources and detectors on the participant's head. An overcap was used to prevent ambient-light contamination. Raw intensities were recorded using the software provided by the manufacturer (NIRStar, version 15.1 and 15.2).

### Data Processing

The data were analyzed using the open-access software Homer 2 within MATLAB (R2019b, Mathworks). The first processing step was to convert raw data into optical density. Then, a low pass filter with a cut-off of 0.1 Hz was applied to attenuate respiration and cardiac activity and high frequency noise (25). The next step was a motion artifact correction using wavelet-based filters (iqr = 1.5) (26, 27). After motion correction, optical density was converted into relative concentration changes of HbO_2_ and HbR using the modified Beer-Lambert law with constant differential path length factors (DPF) values of 6 (28). Finally, contribution of short separation channels was removed from the signal using a Kalman filter dynamic estimator (29). Using the block average method, mean amplitude difference of ΔHbO_2_ and ΔHbR for each channel was obtained using the last 5 s of the resting state before each trial and 30 s after the “start” instruction. Signal quality of each channel was visually checked to ensure divergence between the HbO_2_ and HbR traces (15) and extreme values were detected using the box-plot method. When the number of extreme values were inferior or equal to 3 (≤10%) for a channel, values were replaced by mean values of the group in the specific condition. Otherwise, the channel was removed for all participants. For the subtraction task, C5 and C12 were therefore removed for all participants. Signals from channels were averaged over the PFC (channels 1–8; 11–18), in line with previous research (21). Recommendations and good practices were followed according to a recent consensus paper (30).

### Neuropsychological Tasks

Participants completed a series of tests assessing global cognition (MOCA) (31), inhibition (Stroop test; inhibition cost) (32), psychomotor speed, working memory, and attentional shift [Trail Making Test (TMT) part A and B, TMT (B-A)] (33), attention and processing speed [Digit Symbol Substitution Test (DSST)] (34), mental flexibility (Plus Minus Task; shift cost) (35) and visuospatial abilities (Bells Test) (36). The order of neuropsychological tasks was randomized between participants. They were administered before or after the walking task.

### Statistical Analyses

Kolmogorov-Smirnov tests were used to determine the normality of variables. For demographic, clinical, and neuropsychological variables, between-group differences were examined using Fisher's Exact tests, independent student *t*-tests or Wilcoxon rank-sum tests, as appropriate. For neuropsychological tests, *p*-values were corrected for multiple comparisons using Bonferroni adjustment. A score is significant only if the corresponding *p*-value is ≤0.05/5 (*p* ≤ 0.01).

Repeated measures univariate analyses of variance (ANOVA) with group (patients vs. controls) as between subject factor and condition (standing while subtracting, usual walking and the two DT walking) as within subject factor, and group by condition as interaction effect, were applied on fNIRS data, as recommended in a previous study (37). Dependent variables were ΔHbO_2_ and ΔHbR.

Repeated measures multivariate ANOVA with group as a between subject factor and condition as within subject factor, and group by condition, were applied on gait parameters (gait speed, cadence, stride length and gait cycle time). Dependent variables were mean gait parameters and CV of gait parameters, respectively.

For each ANOVA, effect sizes (η^2^) were reported and were interpreted as small (0.02), medium (0.13), and large (0.26) (38). Bonferroni correction for multiple comparisons was applied during the *post-hoc* analyses.

In all participants, Pearson or Spearman rank correlations were analyzed to investigate associations between cortical activity (ΔHbO_2_) and behavioral performance (gait and neuropsychological outcomes) within condition. Pearson correlations (r) were used when data was distributed normally whereas Spearman rank correlations (ρ) were used when data was not distributed normally. Correlations (ρ or r) were considered weak below 0.10, moderate between 0.10 and 0.49 and strong between 0.50 and 1.00 (39). To determine which factors (age, gender, years of education, BDI, FES-I or/and neuropsychological factors) were the best predictors of HbO_2_ levels during usual walking, stepwise linear regression model was employed with group as a covariate. Only variables that significantly correlated with ΔHbO_2_ during usual walking and did not show strong intercorrelations (*r* < 0.80) were selected for entry in the model. *P* < 0.05 were considered significant. All statistical analyses were conducted using SPSS, version 26.0.

## Results

### Participants

Eighteen patients with early stage PD and 18 controls matched for age, sex and education level were included (Table 1). Patients were categorized into 3 clinical subtypes: 3 patients were classified as postural instability and gait difficulty-predominant disease, 8 patients were classified as tremor-dominant disease, and 7 patients were rigidity-dominant disease. Scores on BDI and FES-I scale were significantly higher in patients with PD, compared to controls. Patients were in early stages of the disease, based on disease duration, the Hoehn and Yahr scale and the motor section of the Unified Parkinson's Disease Rating Scale (UPDRS). Three patients had a Hoehn and Yahr rating at stage 1, 13 were at stage 2, and 2 were at stage 3.

**Table 1:** Demographic and clinical characteristics for all participants.

**Variables**	**PD patients *N* = 18**	**Controls *N* = 18**	***P*-value**
Age, years	68 ± 8	66 ± 7	0.48
Gender (M/F)	11/7	11/7	1.00
Education, years	14.5 ± 3	14.5 ± 2	0.85
MOCA score (0–30)	27.5 (26–29)	27 (26–29)	0.82
BDI score 21 items	6 ± 4	2 ± 2	**0.001**
FES-I score (maximum score, 64)	25 ± 8	19 ± 3	**0.02**
Disease duration, years	5 (2–8)	NA	
Hoehn and Yahr stage (on)	(2–2)	NA	
UPDRS motor (on)	17 (12–26)	NA	
LED, mg/day	604 (310–898)	NA	

*Values are expressed as mean ± SD or median (Q1–Q3). Values in bold are significant. BDI, Beck Depression Inventory; LED, L-Dopa Equivalent Dosage; M, Male; F, Female; FES-I, Falls Efficacy Scale International; MOCA, Montreal Cognitive Assessment; NA, not applicable; UPDRS, Unified Parkinson Disease Rating Scale*.

### Changes in Dorsolateral Prefrontal Cortex Activation

#### ΔHbO_2_ and ΔHbR

##### Group Effect

Patients had higher ΔHbO_2_ than controls [*F*_(1, 34)_ = 7.11, *p* = 0.01, η^2^ = 0.17]. No between-group differences were found for ΔHbR [*F*_(1, 34)_ = 0.54, *p* = 0.47, η^2^ = 0.02].

##### Condition Effect

**Figure 2:**
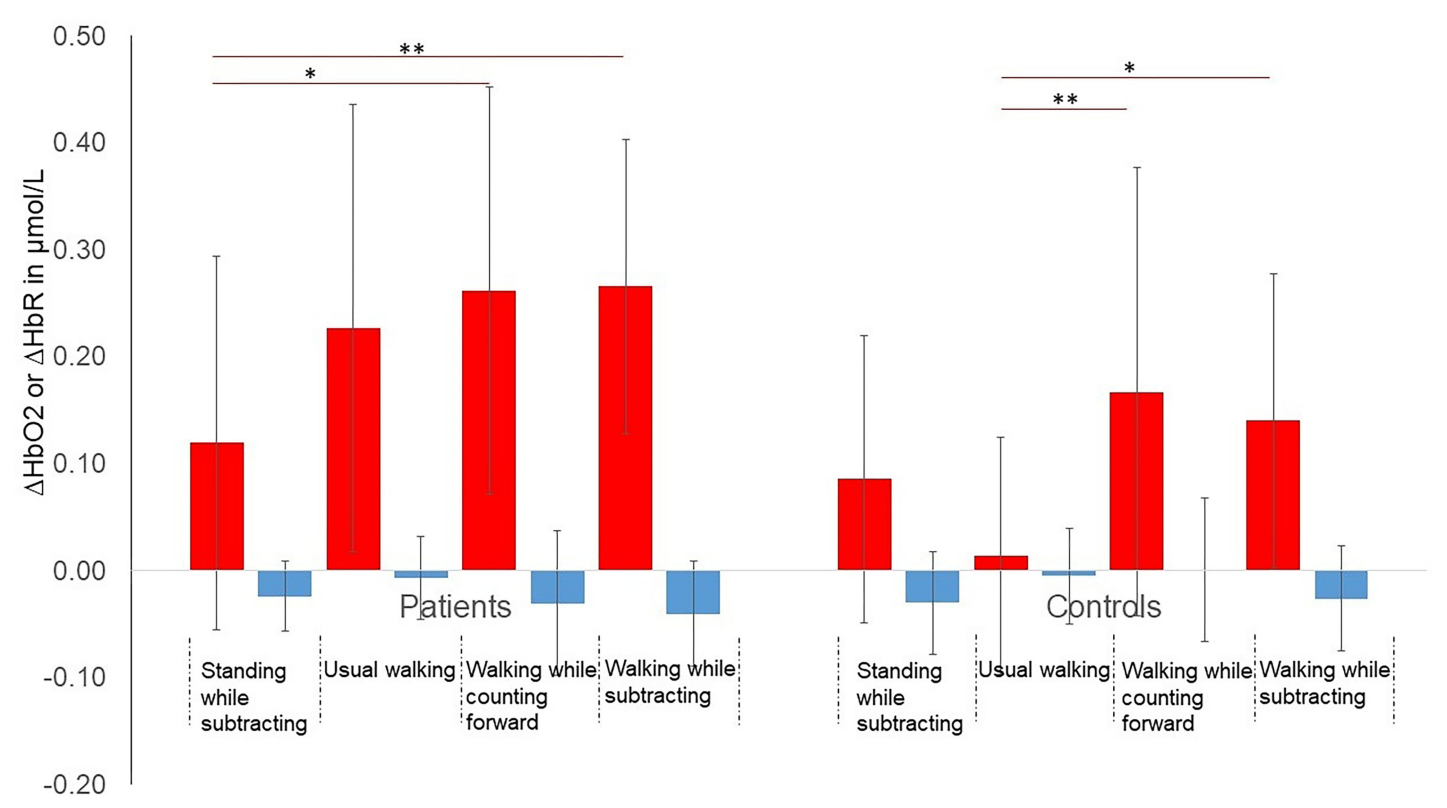
Mean (SD) changes (Δ) in HbO_2_ and HbR during the 4 conditions in both groups. *p < 0.05, **p < 0.01. *P*-values adjusted for Bonferroni correction. Red bars represent ΔHbO_2_ and blue bars represent ΔHbR. Red lines represent significant within-group differences in ΔHbO_2_ between 2 conditions.

Furthermore, significant differences in ΔHbO_2_ and in ΔHbR between conditions [*F*_(3, 102)_ = 7.71, *p* < 0.001, η^2^ = 0.19; *F*_(3, 102)_ = 8.91, *p* < 0.001, η^2^ = 0.21, respectively] were found. ΔHbO_2_ significantly increased during walking while counting forward compared to usual walking (*p* = 0.02). ΔHbO_2_ tended to increase during walking while subtracting condition compared to usual walking (*p* = 0.06). ΔHbO_2_ also increased during both DT walking conditions compared to standing while subtracting condition (*p* < 0.01). ΔHbR significantly decreased during walking while subtracting compared to usual walking (*p* < 0.001) and walking while counting (*p* < 0.001) (Figure 2).

##### Group^*^ Condition Interaction

**Figure 3:**
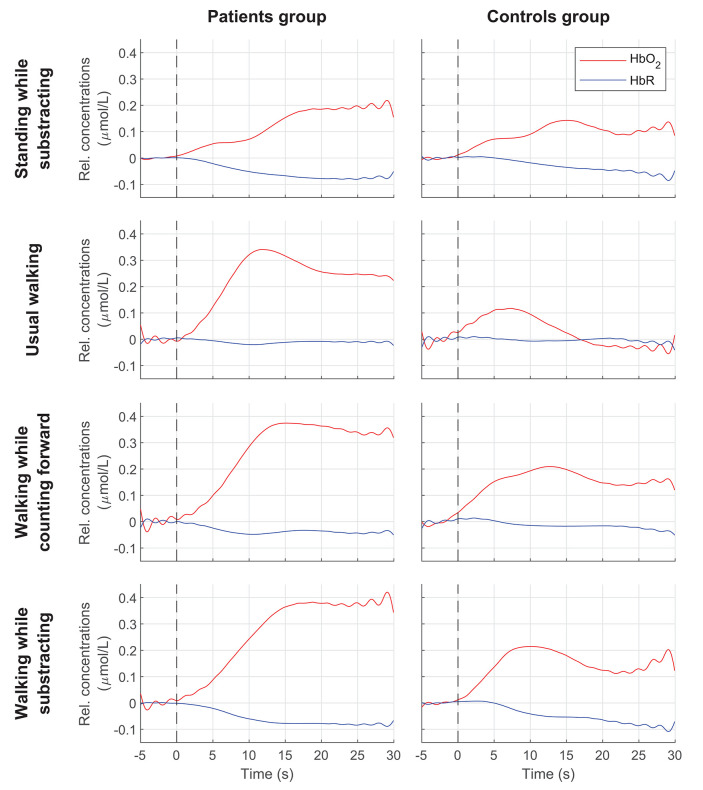
Averaged time courses of HbO_2_ and HbR in the DLPFC for each condition in both groups. Vertical dashed lines indicate start of the task. Rel. concentrations, Relative concentrations.

In addition, the interaction between condition and group on ΔHbO_2_ was significant [*F*_(3, 102)_ = 3.34, *p* = 0.02, η^2^ = 0.09]. Patients had higher ΔHbO_2_ than controls during usual walking (*p* = 0.001) and in DT-walking while subtracting (*p* = 0.02) (Figure 2). In controls, ΔHbO_2_ increased during both DT walking conditions compared to usual walking, which was not the case for the patients (*p* = 1.00). Only in patients, ΔHbO_2_ was significantly higher during the 2 DT walking conditions compared to the standing while subtracting condition. No significant interaction between condition and group was found on ΔHbR [*F*_(3, 102)_ = 1.65, *p* = 0. 18, η^2^ = 0.05]. The Figure 3 shows the averaged time courses of HbO_2_ and HbR in the DLPFC for the 4 conditions in both groups. For all conditions, the average relative concentration changes of HbO_2_ increased after starting the task. The average relative concentration changes of HbR showed slight reductions or remained relatively stable during the task.

### Behavioral Performance

#### Gait Performance

##### Group Effect

Patients had reduced stride length compared to controls (*p* < 0.05) (Table 2).

**Table 2:** Repeated analyses of variance for gait parameters.

**Gait parameters**		**Condition**	**Group**	Condition*Group Pairwise comparisons 1–2; 1–3; 2-3^a^*p*-value^b^
Speed	Mean	***F***_**(2,68)**_ **= 50.08**, ***p <*** **0.001**, **η^2^ = 0.60**	*F*_(1,34)_ = 3.51, *p =* 0.07, η^2^ = 0.09	*F*_(2,68)_ = 1.98, *p =* 0.15, η^2^ = 0.06
	CV	***F***_**(2,68)**_ **= 15.74**, ***p <*** **0.001**, **η^2^ = 0.32**	***F***_**(1,34)**_ **= 4.28**, ***p* = 0.046**, **η^2^ = 0.11**	***F***_**(2,68)**_ **= 3.32**, ***p* = 0.04**, **η^2^ = 0.09** For patients: **0.002; <0.001**; NS For controls: NS; NS; NS
Cadence	Mean	***F***_**(2,68)**_ **= 45.18**, ***p <*** **0.001**, **η^2^ = 0.57**	*F*_(1,34)_ = 0.83, *p =* 0.37, η^2^ = 0.02	*F*_(2,68)_ = 1.43, *p =* 0.25, η^2^ = 0.04
	CV	***F***_**(2,68)**_ **= 20.84**, ***p <*** **0.001**, **η^2^ = 0.38**	*F*_(1,34)_ = 1.06, *p =* 0.31, η^2^ = 0.03	*F*_(2,68)_ = 2.87, *p =* 0.06, η^2^ = 0.08
Stride length	Mean	***F***_**(2,68)**_ **= 29.27**, ***p* < 0.001**, **η^2^ = 0.46**	***F***_**(1,34)**_ **= 4.35**, ***p* = 0.045**, **η^2^ = 0.11**	*F*_(2,68)_ = 2.20, *p =* 0.12, η^2^ = 0.06
	CV	***F***_**(2,68)**_ **= 12.25**, ***p <*** **0.001**, **η^2^ = 0.27**	*F*_(1,34)_ = 2.00, *p =* 0.17, η^2^ = 0.06	*F*_(2,68)_ = 2.35, *p =* 0.10, η^2^ = 0.07
Gait cycle time	Mean	***F***_**(2,68)**_ **= 36.31**, ***p <*** **0.001**, **η^2^ = 0.52**	*F*_(1,34)_ = 1.06, *p =* 0.31, η^2^ = 0.03	*F*_(2,68)_ = 1.72, *p =* 0.19, η^2^ = 0.048
	CV	***F***_**(2,68)**_ **= 16.93**, ***p <*** **0.001**, **η^2^ = 0.33**	*F*_(1,34)_ = 1.20, *p =* 0.28, η^2^ = 0.03	***F***_**(2,68)**_ **= 5.38**, ***p* = 0.007**, **η^2^ = 0.14** For patients: **0.001; <0.001; 0.001** For controls: **0.03;** NS; NS

*Values in bold are significant*.

a *(1) Usual walking; (2) Walking while counting forward; (3) Walking while subtracting*.

b *Adjusted for multiple comparisons using Bonferroni correction. NS, Not Significant; CV, Coefficient of variation*.

##### Condition Effect

Speed, cadence, stride length, as well as gait cycle time were altered as the walking task difficulty increased (see Table 2). CV of cadence, speed and gait cycle time were greater during the 2 DT walking conditions than during the usual walking (CV of cadence, speed, and gait cycle time, *p* ≤ 0.001). CV of stride length was greater during walking while subtracting condition than during walking while counting (*p* = 0.04) or during usual walking (*p* < 0.001).

##### Group^*^Condition Interaction

**Figure 4:**
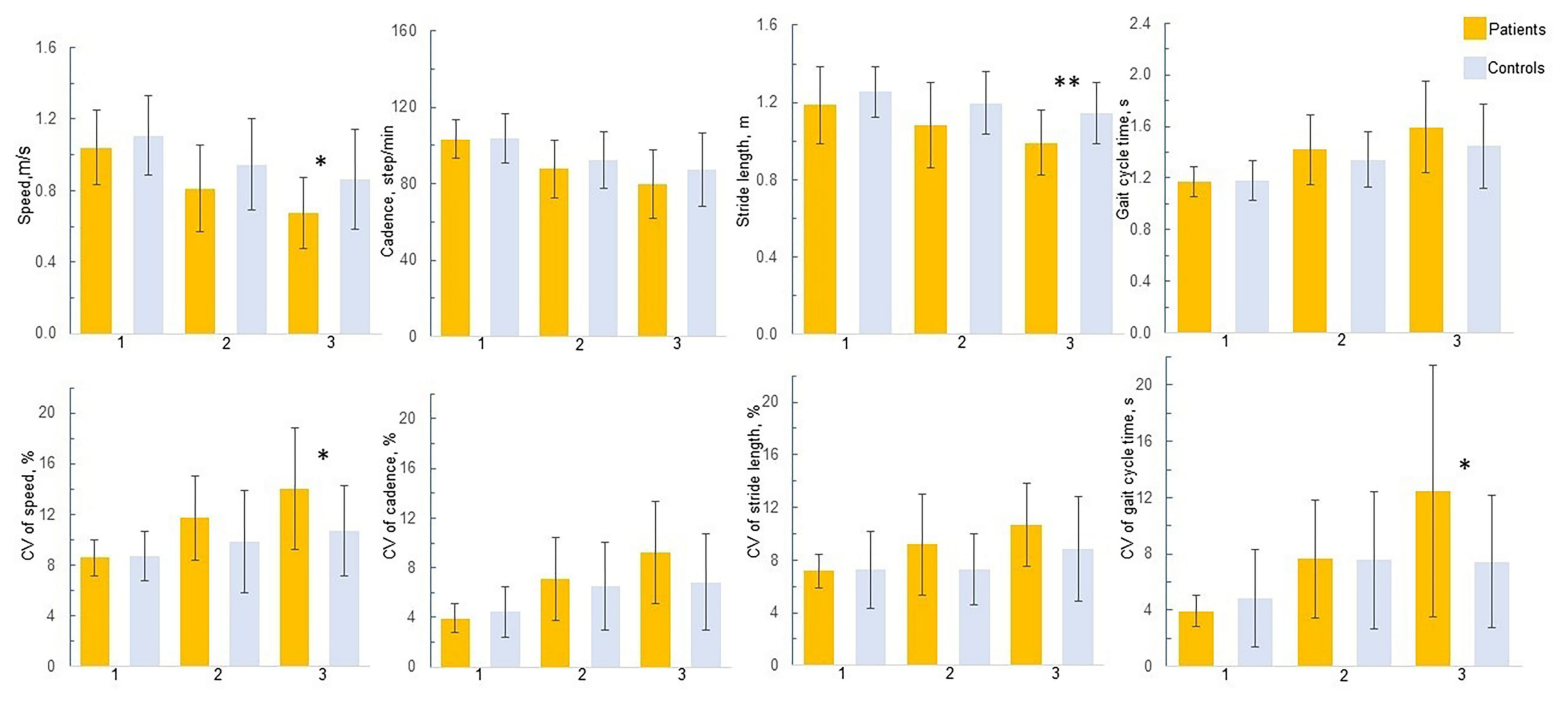
Gait performance in patients and controls. 1 = Usual walking; 2 = Walking while counting forward; 3 = Walking while subtracting. *p < 0.05, **p < 0.01; *P*-values adjusted for Bonferroni correction. Only significant between-group differences are presented in the figure.

No interaction between group and condition was found in any of the mean gait parameters (*p* > 0.12) (Table 2). However, two interactions between group and condition on CV of speed (*p* = 0.04) and CV of gait cycle time (*p* = 0.007) were significant. In patients, CV of speed increased during both DT walking conditions compared to usual walking whereas no pairwise comparisons for the CV of speed were found for the controls. Furthermore, patients had greater CV of speed than controls during the walking while subtracting condition (*p* = 0.02) (Figure 4). In patients, CV of gait cycle time increased as walking task difficulty increased whereas in controls, CV of gait cycle time increased during DT walking while counting forward compared to usual walking (*p* = 0.03). Patients also had a greater CV of gait cycle time than controls during the DT walking while subtracting (*p* = 0.04).

### Cognitive Performance

No significant between-group differences in cognitive performance were found during the standing while subtracting and walking while subtracting conditions (standing while subtracting: Controls: 5.61 ± 3.19; patients: 5.91 ± 3.22, *p* = 0.83; walking while subtracting: Controls: 4.85 ± 2.74; patients: 5.28 ± 3.11, *p* = 0.67). Patients were significantly better than controls in the walking while counting forward condition (27.31 ± 5.60 vs. 22.42 ± 7.17, *p* = 0.02).

Patients were significantly slower than controls to perform the Stroop test (word reading conditions), the TMT (part A and B), and the PMT (the addition list) (see Table 3). They performed worse than controls on the DSST.

**Table 3:** Comparison of performance on neuropsychological tests between PD patients and controls.

	**PD patients *N* = 18**	**Controls *N* = 18**	***P*-value^b^**
Stroop color, s (1)	68.00 (62.00–75.00)	58.50 (54.00–72.00)	0.03
Stroop word, s	48.00 (45.00–53.00)	42.00 (38.00–46.00)	**0.002**
Stroop C/W, s (2)	132.50 (108.00–140.00)	102.50 (89.00–135.00)	0.06
Inhibition cost, s (2)–(1)	55.50 (47.00–74.00)	45.00 (33.00–58.00)	0.24
TMT A, s (3)	53.50 (44.00–72.00)	40.50 (35.00–45.00)	**0.009**
TMT B, s (4)	119.00 (94.00–157.00)	67.50 (60.00–88.00)	**0.002**
TMT (B-A), s (4)–(3)	67.50 (50.00–83.00)	29.50 (25.00–44.00)	**0.004**
DSST, score	50.11 ± 14.00	66.78 ± 16.32	**0.002**
PMT—addition list, s (5)	68.00 (54.00–78.00)	50.50 (43.00–58.00)	**0.006**
PMT—subtraction list, s (6)	102.50 (74.00–117.00)	74.00 (54.00–100.00)	0.04
PMT—alternance list, s (7)	109.50 (87.00–133.00)	78.00 (72.00–101.00)	0.05
PMT—shift cost, s^a^	18.25 (9.00–28.50)	18.75 (10.50–29.00)	0.85
Bells, score	34.00 (33.00–35.00)	34.00 (33.00–35.00)	0.48
Bells, time	153.13 ± 38.11	137.89 ± 51.52	0.31

*Values are expressed in mean ± SD or median (Q1–Q3). Values in bold are significant*.

a (7) – [(6) +(5)]/2

b *adjusted for Bonferroni correction*.

*DSST, Digit Symbol Substitution Test; PMT, Plus-Minus Task; Stroop C/W, Color-Word condition of the Stroop Test; TMT, Trail Making Test*.

### Correlations Between ΔHbO_2_ and Behavioral Measures

Strong correlations between ΔHbO_2_ during usual walking and CV of cadence, stride length and gait cycle time were found (Table 4). Moderate correlations were also found between ΔHbO_2_ during walking while counting forward and CV of all gait parameters. No significant correlations between ΔHbO_2_ during walking while subtracting condition and gait outcomes were found. A higher number of significant correlations between ΔHbO_2_ and cognitive performance was found for the usual walking condition than for the other conditions. Increased ΔHbO_2_ during usual walking was negatively correlated with worse performance on TMT, DSST, and Stroop test (color and word conditions). Note that no significant correlations between ΔHbO_2_ and behavioral measures were found when conducting correlation analyses in each group (data available upon request).

**Table 4:** Associations between ΔHbO_2_ and behavioral performance.

			**HbO_**2**_**		
		**Standing while subtracting**	**Usual walking**	**Walking while counting forward**	**Walking while subtracting**
Speed	Mean	NA	−0.23	−0.04	−0.09
	CV (%)		−0.008	0.45^***^	0.25
Cadence	Mean		−0.05	−0.01	0.006
	CV (%)		***−0.12***	−0.39^*^	0.11
Stride length	Mean		−0.28	−0.05	−0.14
	CV (%)		***0.18***	−0.50^**^	0.14
Gait cycle time	Mean		0.06	0.08	0.06
	CV (%)		***−0.11***	0.42^**^	0.18
MOCA score		−0.01	−0.17	0.04	−0.04
Stroop color		0.26	0.40^*^	0.27	0.33
Stroop word		0.36^*^	0.35^*^	0.24	0.33
Stroop C/W		0.22	0.24	0.12	0.16
Inhibition cost		*0.09*	*−0.18*	*−0.06*	*0.08*
TMT A		*0.31*	0.55^***^	0.33^*^	0.46^**^
TMT B		*0.11*	0.64^***^	*0.29*	0.39^*^
TMT (B-A)		*−0.08*	0.62^***^	*0.25*	*0.33*
DSST		−0.04	−0.40^*^	−0.11	−0.33^*^
PMT—addition list		0.11	0.16	0.07	0.17
PMT—subtraction list		0.19	0.10	0.24	0.03
PMT—alternance		0.21	0.20	0.07	0.22
PMT—shift cost		0.14	0.18	0.06	0.09
Bells score		*−0.15*	*−0.10*	*−0.08*	*−0.27*
Bells time		0.30	0.30	0.12	0.18

*The values correspond to Pearson correlations except for the ones in italics which represent Spearman correlations. Values in bold are significant*.

* *p < 0.05*;

** *p < 0.01*;

*** *p < 0.001. DSST, Digit Symbol Substitution Test; NA, Not Applicable; PMT, Plus-Minus Task; TMT, Trail Making Test*.

### Linear Regression Analysis

Among demographic variables, age and years of education were also significantly correlated with ΔHbO_2_ during usual walking (age: *r* = 0.37, *p* = 0.03; level of education: *r* = −0.39, *p* = 0.02).

Age, years of education, time to complete the color condition of the Stroop test, times to complete part A and B of the TMT, CV of cadence, CV of stride length were included in the model, with the group as a covariate. Stepwise linear regression revealed that part B of TMT was the most significant predictor of ΔHbO_2_ during usual walking. This test explained 21% of the variance. Years of education was then introduced in addition to part B of TMT, explaining an additional 6% of the variance (Table 5). Note that part B of TMT was the most significant predictor, explaining 27% of the variance when the stepwise linear regression was performed in PD patients only.

**Table 5:** Predictors of ΔHbO_2_ during usual walking.

**Independent variables included in the model**	***R*^**2**^**	***R^**2**^ change***	***B***	***t***	***P*-value**
**Step 1**					
Group^a^	0.29	0.29	0.21	3.66	**<0.001**
**Step 2**					
Group	0.49	0.21	0.13	2.29	**0.03**
TMT-B			0.001	3.60	**0.001**
**Step 3**					
Group	0.56	0.06	0.14	2.67	**0.01**
TMT- B			0.001	2.91	**0.007**
Years of education			−0.02	−2.08	**0.04**

*ΔHbO2 was the dependent variable. Values in bold are significant*.

a *Included in the model as a covariate*.

## Discussion

In this study, we aimed to investigate changes in cortical activity of the DLPFC during usual walking and DT walking conditions in patients with PD in comparison with controls. Associations between cortical activity in the DLPFC during usual and DT walking and behavioral/cognitive outcomes were also examined. A third objective was to determine the best predictors of an increased activation in the DLPFC during usual walking. The novelty of this research is that we further explored the determinants of an increased cortical activity during usual walking. This may help to better understand falls risk or gait deficits before they emerge.

### Increased Brain Activity in DLPFC in Patients With PD During Walking

#### Usual Walking

Moderate to strong associations were found during usual walking between increased cortical activity and poorer performance in neuropsychological tests assessing executive functions, speed of processing and psychomotor speed (i.e., TMT, DSST, and color and word conditions of the Stroop test). These findings suggest that normal walking is already a complex process that requires input from executive functions (40), that is carried out by DLPFC with other projections to various cortical and subcortical regions (41). As expected, patients had executive deficits and processing speed slowness compared to controls.

Patients with PD had also higher levels of DLPFC activation in comparison with controls, even during usual walking, which is consistent with previous studies (6, 11, 13, 21, 42, 43). However, patients did not show decreased walking performance compared to controls during usual walking. These results are not consistent with previous studies reporting decreased walking performance in patients already during the usual walking condition (20, 44). An explanation could be due to clinical and demographic characteristics of our group of patients. Our patients were at the earliest stages of PD, based on disease duration and motor score of UPDRS. They were also younger than those recruited in previous studies (20, 44). Furthermore, they were all volunteers to participate in this study and physically active. As in most research, there is a recruitment bias: it is often the fittest patients who agree to participate. It is therefore not excluded that these patients represent a special category, not quite representative of PD patients' population. It is also possible that our path configuration with markings on the floor helped patients to walk. Other measures, such as measures of arm swing while walking could be used in future studies, to better discriminate patients with PD from controls. Indeed, this measure has been shown as a new prodomal marker of PD (45).

Finally, this increased cortical activity in the DLPFC in absence of gait impairments during usual walking may reflect a compensatory mechanism to overcome executive deficits, processing speed slowness and deficits in movement automaticity in early stage PD (46).

#### DT Walking

No significant increase of DLPFC activity in patients was found from usual walking to DT walking conditions whereas in controls, significant increase appeared from usual walking to DT walking conditions. Findings on patients are consistent with previous studies that used the same type of DT walking (20). These results suggest that patients are unable to further increase DLPFC activity, due to a reduction of their cognitive resources and their limited cognitive capacity. Results in controls are consistent with previous studies reporting a significant increase in PFC, as a compensatory mechanism in healthy older adults during DT walking (e.g., walking while serially subtracting 3s) (47–50). This is also in line with the cognitive resource theories of aging which postulated that brain activity will be more pronounced with increasing task demands in older adults (51). Increased prefrontal activity in older adults may also be due to the fact that most of healthy older adults in the present study were younger than 70s. A recent study suggested that healthy older adults in the 70s were unable to further increase prefrontal cortex activity, leading to a decrease of walking performance (52).

During walking while counting forward, no significant between-group differences were observed for DLPFC activity and gait measures. Furthermore, patients performed better in the counting forward condition while walking than controls. It is possible that patients prioritize the counting task at the expense of the walking task. Patients are globally slower, with a reduced cadence and shorter stride length than controls during this condition although no significant between-group differences in gait performance were found. Another explanation could be that patients had sufficient cognitive resources to perform both the counting forward condition and the walking task. Association between cortical activity in the DLPFC and cognitive measures revealed that increased DLPFC activity during walking while counting forward condition is rather associated with psychomotor slowness than executive functions. This result also highlights the major role of this cortical area on psychomotor functioning (53). The lack of significant differences between groups in cortical activity and gait measures may also be due to a high heterogeneity in the performance.

Patients with PD had higher levels of DLPFC activation than controls during walking while subtracting condition. As expected, increased DLPFC activity during walking while subtracting condition was found to be associated with executive deficits and psychomotor slowness. Furthermore, patients performed as well as controls the subtraction task while walking. However, they walked slower and had shorter stride length than controls. Patients were also more irregular than controls, as reflected by a greater variability of speed and gait cycle time. Based on the concept that attentional capacity is limited, it is possible that patients allocated their available cognitive resources to the subtraction task at the expense of the walking task. Increased DLPFC activation in patients during this complex DT condition is therefore insufficient to compensate for motor and executive deficits and may lead to decreased walking performance (12).

### Associations Between Cortical Activity and Gait Parameters

Associations between increased activation in the DLPFC and higher gait variability in all participants were already found during usual walking. Similar findings were found in previous studies in older adults during obstacle negotiation (48). These authors discussed the fact that increased activation in the DLPFC may reflect a compensatory attempt to overcome inefficient neural activation in the face of demanding tasks. The inefficiency was unable to cope with the motor needs. During the walking while counting forward condition, increased cortical activity was associated with lower variability of their cadence and stride length as well as a greater variability of speed and gait cycle time. A possible explanation of the lower variability of cadence and stride length is that counting forward may induce a rhythm on which participants paced their walking. However, higher variability in their speed and gait cycle time seems to be moderately associated with increased cortical activity in the DLPFC during walking while counting forward. The lack of significant correlations between HbO_2_ and gait parameters in the DT walking while subtracting condition could be explained by the fact that patients with PD are unable to further increase their cortical activity in the DLPFC.

In the literature, measures of gait variability were found to be closely related to instability and fall risk (54). Strong relationships between high gait variability and increased cortical activity during usual walking supports the notion that subtle variations in the gait during usual walking, particularly in older individuals with PD, may precede disturbances in DT walking, leading to a higher risk of falls. To our knowledge, few studies explored associations between cortical activity and behavioral measures in walking tasks in older individuals with or without PD (52, 55). Further studies with larger sample size are needed to better understand the relationships between cortical activity and gait parameters in this population.

### Best Predictors of Increased Cortical Activity During Usual Walking

Poorer performances on part B of TMT, and a low level of education significantly contributed to explain increased cortical activity in the DLPFC during usual walking. Particularly, the TMT-B explained 21% of the variance after adjusting for group status. These findings highlight the major role of TMT-B in predicting cortical activity of the DLPFC during usual walking. This complex test requires executive functions (updating information in working memory, shifting attention between letters and number as well as inhibit non-relevant information), which is affected by PD. This test in clinic may prove useful to determine difficulties in activities of daily living in patients with PD, as previously reported in a driving context (56). Yet, caution with the interpretation is warranted as the sample of the study was relatively small. Finally, a lower level of education also contributed to increased cortical activity in the DLPFC which is in accordance with the evidence that better executive functions are related to an increased number of education years (57). Further studies should therefore consider the level of education of patients with PD when exploring changes in brain activity in the DLPFC.

### Methodological Considerations

Methodological considerations of this study included the use of short-separation reference channels (1.5 cm apart) to remove for peripheral hemodynamic response (i.e., artifact caused by breathing, cardiac cycle or other error related to movement) which has been used only once in patients with PD (15). Furthermore, both Hb species were reported. ΔHbO_2_ was found to be the most sensitive indicator of regional cerebral blood flow in NIRS measurements (58) whereas ΔHbR better reflects the match between oxygen supply and demand (59). In this study, fNIRS processing was the same for patients with PD and healthy older individuals. Although a recent study showed few effects of different processing methods on fNIRS signals assessed during active walking tasks in older adults (60), future studies applying specific fNIRS processing (e.g., age-dependent DPF values) are warranted. Furthermore, it would have been interesting to include a standing while counting forward condition in order to determine different patterns of task prioritization between groups (61). However, as the duration of the experiment was already long, we preferred to limit the number of conditions. The small sample size including volunteers with well-preserved cognitive abilities among PD participants limits the generalizability of the results. One limitation of the present study is that only the activity of DLPFC cortex is recorded. Further studies should assess multiple cognitive regions while walking to provide a greater understanding of the contribution of brain areas to walking and dual tasking (62). Finally, except the fear-of-falling inventory, no direct measures on the number of falls in patients with PD was reported in this study. Further studies should include a detailed questionnaire reporting the number of falls.

### Clinical Implications

These findings may have important clinical implications for training program: it is possible that cognitive intervention decreases DLPFC activity and therefore improve the ability to walk in usual and complex situations. However, the increased cortical activity in the DLPFC is also due to deficits in automaticity (46). Multimodal intervention that targets both motor and cognitive aspects should therefore be developed to improve cognition and movement automaticity and promote mobility in individuals with PD. As a consequence, a reduction in the DLPFC activity while walking after the intervention should be expected. Further studies investigating the effect of training programs on cortical activity in DLPFC in patients with PD are therefore warranted.

## Conclusions

This present study shows that patients with early stage PD increased their DLPFC activity, already during usual walking to compensate for subcortical dysfunction and executive deficits. Furthermore, strong relationships between cortical activity in the DLPFC during usual walking and behavioral measures were found in all participants. Finally, longer times to complete part B of TMT, and lower number of education years were found to be the best predictors of an increased of ΔHbO_2_ during usual walking.

## Data Availability Statement

The raw data supporting the conclusions of this article will be made available by the authors, without undue reservation.

## Ethics Statement

The studies involving human participants were reviewed and approved by Comité de Protection des Personnes Nord Ouest III Réf. CPP: 2018-01 No. ID RCB: 2017-A03187-46. All participants provided their written informed consent to participate in this study.

## Author Contributions

MR, IH, MC, JL, TD, and LP-F conceived and designed the study. MR, IH, MC, RD, TD, and LP-F contributed to data collection. MR, IH, RD, and LP-F contributed to data analysis and interpretation. IH, MC, RD, HD, SP, JL, TD, and LP-F contributed to the critical revision for important intellectual content. MR wrote the first draft. All authors contributed to the article and approved the submitted version.

## Conflict of Interest

The authors declare that the research was conducted in the absence of any commercial or financial relationships that could be construed as a potential conflict of interest.
